# Large-scale decrease in the social salience of climate change during the COVID-19 pandemic

**DOI:** 10.1371/journal.pone.0256082

**Published:** 2022-01-19

**Authors:** Brian R. Spisak, Bogdan State, Ingrid van de Leemput, Marten Scheffer, Yuwei Liu

**Affiliations:** 1 Harvard University, Cambridge, MA, United States of America; 2 Vrije Universiteit Amsterdam, Amsterdam, The Netherlands; 3 Scie.nz, Wellington, New Zealand; 4 Victoria University of Wellington, Wellington, New Zealand; 5 Wageningen University & Research, Wageningen, The Netherlands; 6 Independent Researcher, United States of America; University of Haifa, ISRAEL

## Abstract

There are concerns that climate change attention is waning as competing global threats intensify. To investigate this possibility, we analyzed *all* link shares and reshares on Meta’s Facebook platform (e.g., shares and reshares of news articles) in the United States from August 2019 to December 2020 (containing billions of aggregated and de-identified shares and reshares). We then identified all link shares and reshares on “climate change” and “global warming” from this repository to develop a social media salience index–the Climate SMSI score–and found an 80% decrease in climate change content sharing and resharing as COVID-19 spread during the spring of 2020. Climate change salience then briefly rebounded in the autumn of 2020 during a period of record-setting wildfires and droughts in the United States before returning to low content sharing and resharing levels. This fluctuating pattern suggests new climate communication strategies–focused on “systemic sustainability”–are necessary in an age of competing global crises.

## Introduction

Climate change and COVID-19 represent two global crises unfolding on different time scales, and there are growing concerns that immediate threats such as COVID-19 are taking public interest away from the long-term challenges of climate change (i.e., the “distraction effect”; [1]). Indeed, research indicates that humans have finite supplies of “surplus compassion” and limited “carrying capacity” for multiple mass communication topics due to cognitive limitations [2]. Mapping the social salience of climate change (i.e., the topic’s prominence in society) is therefore germane across disciplines–from social influence research in economics and psychology [3, 4] to the exploration of social tipping points and synchronization in complex systems research and network science [5, 6]. Large-scale social salience insights also contribute to important practical goals such as predicting and managing fluctuations in “moral indignation, political celebration, ideological fervor, happiness, and value judgments” (p. 1411; [7]).

However, though large-scale climate change salience is an important concern, especially when threats such as COVID-19 emerge and potentially compete for attention [1], there is little to no empirical research on the topic given the chronic problem of limited access to (high-quality) large-scale temporal data [8]. Here, we address this shortcoming using the largest known dataset capturing climate change salience. Specifically, to explore whether the spread of the pandemic is associated with shifts in large-scale climate change salience, our analysis draws upon the total number of link shares and reshares on Meta’s Facebook Platform (e.g., shares and reshares of news articles) in the United States from August 2019 to December 2020 (containing billions of aggregated and de-identified shares and reshares).

This is a novel and robust approach compared to explicit measures of salience (e.g., surveys) which can lead to biases such as social desirability. For example, participants may report high levels of climate change concern because it is perceived as socially favorable, but their actual level of engagement is low. Instead, our approach “passively” measures *actual* large-scale behavior across a diverse, near population-level sample.

## Materials and methods

We downloaded the data through the Data for Good program at Meta. The data is available to academics and nonprofits through the Meta Data for Good Data License Agreement (for contact information see: https://dataforgood.fb.com/tools/climate-conversation-maps/). To create the data, Meta pulled the daily volume of link shares and reshares in 21 languages. The 21 languages were selected with the consideration of both the ground population and the number of active Meta users using them on Meta’s Facebook platform. The languages were English, Spanish, French, Arabic, Portuguese, Hindi, Russian, Japanese, Filipino, Vietnamese, German, Turkish, Burmese, Korean, Italian, Thai, Indonesian, Bengali, Romanian, Chinese, and Polish. We subsequently decided to use all available languages in the dataset given the diversity of the United States. Also, though the data in the entire repository is global, we focused on the United States given the large percentage of Facebook platform users in the country *and* the intensity of the pandemic in the region.

The subset of shares and reshares of links that contained in their title or blurb the keywords “climate change” or “global warming” in those languages were flagged as “climate (re)shares”. The volume of climate link shares and reshares were aggregated on the Global Administrative Areas Database (GADM; http://www.gadm.org/) level 1 admin polygons (US state-equivalent) based on the predicted home location of the (re)sharers provided by Meta. In addition to volume, we also calculated the percentage of climate link shares and reshares relative to total link shares and reshares.

The repository dates to August 2019 and updates daily. For more information including the methodology Meta uses to make the data available for research, please refer to the dataset page from Data for Good at Meta (https://dataforgood.fb.com/tools/climate-conversation-maps/). The code used to analyze the data is freely available upon request and the research was approved by Meta internal review (for an overview of the composition and remit of research review at Meta see: https://research.fb.com/blog/2016/06/research-review-at-facebook/).

Several steps were also taken for privacy preservation when Meta created the dataset. First, only the shares and reshares of links–not original posts–were counted in the data as climate (re)shares. Second polygons having less than ten unique users (re)sharing climate change content were filtered out. Third, only aggregated and anonymized link shares and reshares are available in the dataset (i.e., Meta removed individual-level, personal data to avoid any threat to personal privacy).

## Results and discussion

We evaluated changes in the salience of climate change in the United States through a “Climate Social Media Salience Index” (Climate SMSI). The Climate SMSI is constructed as the ratio between all Facebook link shares and reshares containing “climate change” or “global warming” relatively to the total number of *all* Facebook link shares and reshares made in a certain region in a day.

**Fig 1:**
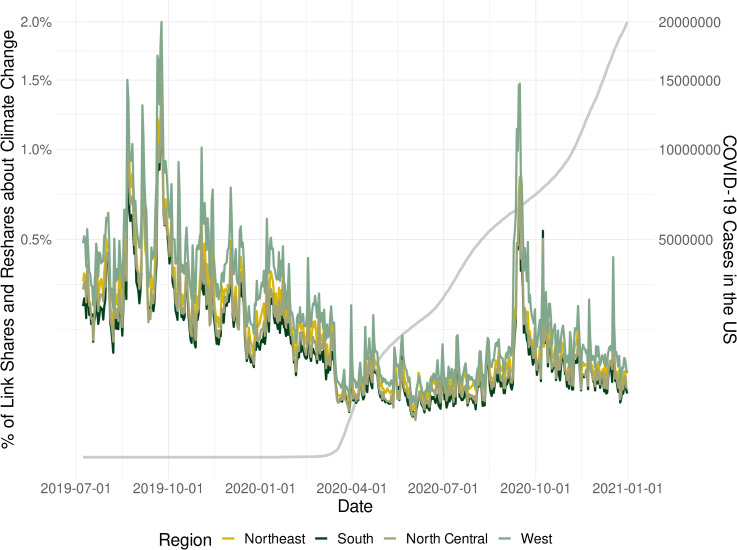
Percentage of climate change link shares and reshares relative to all shares and reshares in the United States.

Fig 1, shown below, plots the Climate SMSI for all US macro-regions over the time frame provided by the extracted dataset. Shown in gray is the cumulative number of COVID-19 cases in the United States as reported by The New York Times [9]. The plot shows a clear shift in link shares and reshares related to climate change as the pressure of COVID-19 increased during March 2020. The shift was staggering. If we look at August 1, 2019 to March 23rd, 2020, we saw a median of 0.25% of all daily Facebook link shares and reshares by US users referencing climate change. The median during the period March 24th to August 8 was only 0.05%–an 80% drop in link shares and reshares related to climate change. The data also shows uniform shifts across US regions: from 0.37% to 0.07% in the Western United States, 0.26% to 0.06% in the Northeast, 0.23% to 0.04% in the North Central Region, and 0.21% to 0.04% in the South. Interestingly, there is also a second spike in climate link shares and reshares during the autumn of 2020. This is likely the result of a record-setting wildfire season as well as extreme heat waves and droughts–particularly in the Western United States–increasing the salience of climate change [10]. Finally, though all regions experienced a second climate salience spike, it is interesting to note that it was largest in the West (i.e., the light green line in Fig 1). This is perhaps due to the region’s proximity to the threat.

**Fig 2:**
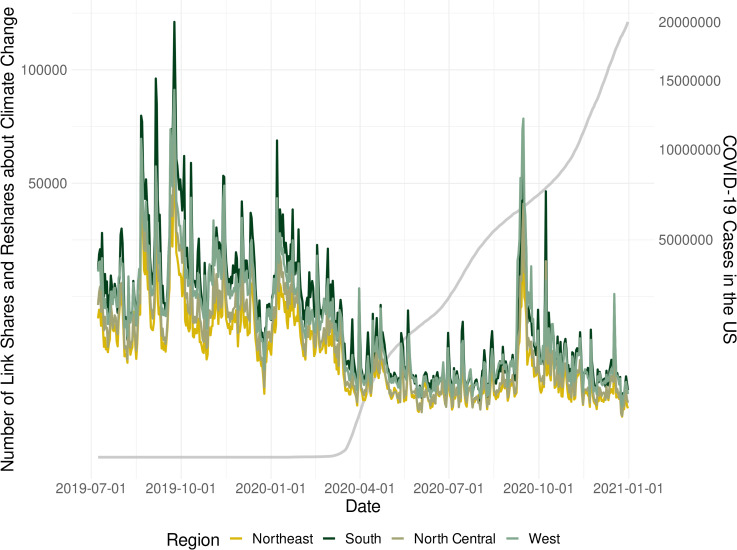
Absolute number of climate change link shares and reshares in the United States.

We also compared our relative Climate SMSI score (i.e., climate change link shares and reshares over all link shares and reshares) to an absolute measure of just the number of climate change link shares and reshares on Facebook in the United States during our time frame of interest. We conducted this analysis to explore whether climate change salience actually decreased or if attention to climate change remained the same and the overall number of Facebook link shares and reshares simply increased due to a content boost from the pandemic. As seen in Fig 2, the absolute measure of climate change salience is similar to the Climate SMSI score. In both measures, there is a clear decrease in climate change link shares and reshares during the outbreak of the pandemic and a rebound in the autumn of 2020.

Thus, our findings are an important step for understanding large-scale shifts in relative *and* absolute climate change salience. For example, this study captured the tectonic decrease in large-scale climate change salience during the COVID-19 outbreak which may otherwise go overlooked using traditional survey methods (e.g., the social desirability problem mentioned above where respondents report climate concern but engage very little with the topic). The time series data showed a clear decrease in Facebook link shares and reshares related to climate change as the pressure of COVID-19 increased during March 2020. Again, the shift was staggering in both absolute climate change link shares and reshares as well as climate change link shares and reshares relative to all content shared and reshared (i.e., the Climate SMSI). Collectively, these measures suggest climate change salience can dramatically increase and decrease as global threats ripple through society.

This salience shifting implies that climate change (as a standalone social topic) is competing with other issues vying for large-scale attention. Scholars therefore need to consider how perturbations such as pandemics, civil unrest, and financial crises interact and interfere with large-scale climate change salience. Developing models of green behavior and transitions, for instance, will suffer from decreased accuracy without such considerations. A better understanding of large-scale climate change salience also has practical implications. Practitioners may benefit from aligning multiple global challenges such as COVID-19 and climate change to avoid issue competition [11].

As for future work, researchers should start exploring causal mechanisms and the complex feedback loop between media producers and consumers. For example, media outlets may produce less climate content to consume as large-scale attention shifts towards an alternative threat such as a pandemic and vice versa (i.e., consumers share less climate content, producers provide less content to share, or both). Also, our data focused on the social salience of climate change over time. Though this provides valuable information regarding broad engagement with climate change–noting that greater engagement can lead to greater acceptance of climate science [12]–researchers will want to investigate the valence (i.e., positive and negative orientations) of large-scale salience. Looking at salience says something about the trend of attention in society while valence relates to nuanced social factors such as voting behavior. In short, looking at salience and valence respectively asks if something is grabbing society’s attention and in what direction.

## Conclusion

The aim of the present study was to investigate possible shifts in large-scale climate change salience during the spread of COVID-19. The primary question was whether populations stay focused on climate change when competing global threats emerge. Our results suggest that climate change salience can indeed fluctuate when there are multiple mass communication topics on the public agenda. Investigating the dynamics of large-scale salience is therefore an important step for ensuring climate change is not lost in a sea of global threats.

If society is continually distracted with seemingly independent shocks from pandemics, civil unrest, and financial crises, then efforts to introduce lasting climate action will suffer. Instead, scholars and practitioners must communicate the interconnectedness between these problems to create a unified message of “systemic sustainability”. Society needs to appreciate how diverse challenges like the loss of biodiversity, pandemics, hyper-urbanization, and extreme concentrations of wealth are connected. The outcome is consistent large-scale interest in climate change–rooted in systemic sustainability–rather than fluctuating and competing spikes in attention over time.
